# Mental and physical health correlates of the psychological impact of the first wave of COVID-19 among general population of Pakistan

**DOI:** 10.3389/fpsyg.2022.942108

**Published:** 2022-10-19

**Authors:** Syed Messum Ali Kazmi, Christopher Alan Lewis, Syeda Salma Hasan, Rabia Iftikhar, Muhammad Umar Fayyaz, Fayyaz Ahmed Anjum

**Affiliations:** ^1^Department of Higher Education, Government of the Punjab, Lahore, Pakistan; ^2^School of Psychology and Therapeutic Studies, Faculty of Social and Health Sciences, Leeds Trinity University, Leeds, United Kingdom; ^3^Department of Psychology, Government College University, Lahore, Pakistan; ^4^Department of Clinical Psychology, Government College University, Lahore, Pakistan; ^5^Department of Psychology, National University of Medical Sciences, Rawalpindi, Pakistan; ^6^Department of Psychology, Preston University, Islamabad, Pakistan

**Keywords:** mental health, physical health, first wave of COVID-19, stress, anxiety, depression

## Abstract

The primary aim was to assess the role of mental and physical health of COVID-19 and its psychological impact in the general population of Pakistan during the first wave of COVID-19. It was hypothesized that there would be a significant predictive association among socio-demographic variables, psychological impact and mental health status resulting from COVID-19, and poor self-reported physical health would be significantly associated with adverse psychological impact and poor mental health status because of COVID-19. A cross-sectional survey research design was used in which 1,361 respondents were sampled online during lockdown imposed in the country. The Impact of Events Scale-Revised (IES-R) was used to assess the psychological impact of COVID-19, and the Depression Anxiety Stress Scales (DASS-21) was used to assess participants’ mental health status. 18% of the respondents reported moderate to severe event-specific distress, 22.6% reported moderate to severely extreme depression, 29% reported moderate to extreme anxiety, and 12.1% reported moderate to extreme stress. Female gender, having graduate-level education, currently studying, and self-reported physical symptoms (persistent fever, chills, headache, cough, breathing difficulty, dizziness, and sore throat) were significantly associated with higher levels of psychological impact exhibited through higher scores on the IES-R and poorer mental health status exhibited through higher scores on the DASS-21 (Depression, Anxiety, and Stress Subscales).

## Introduction

In December 2019, a severe acute respiratory illness, referred to as Corona Virus (COVID-19), originated in Wuhan, Hubei Province, China. Since then, the disease rapidly expanded to almost all countries in the world (Zhou et al., 2020; Zu et al., 2020). Initially, the disease was referred as an epidemic primarily since impact of the disease was initially limited to China, Iran, and a few other countries. On 11th January 2020 China documented its first death from COVID 19. As of 31st March 2020, about 785,000 cases have been reported worldwide, with almost 37,820 deaths (WorldoMeter, 2020). COVID 19 was declared a pandemic by the World Health Organization on 11th March 2020 (Lai et al., 2020). Research has also shown that the initial pace of transmission was initially slow, but in the last 10 days of March 2020, the pace increased to almost 70,000 to 900,00 cases per day (World Health Organization, 2020).

COVID-19 is an infectious disease that has resulted from a new virus. The disease causes a respiratory illness with many symptoms, including cough, flu, persistent fever and, in more severe cases, difficulty in breathing (Rodríguez-Rey et al., 2020b; Russell et al., 2020). Researchers have documented it as an extension of SARs (severe acute respiratory syndrome), due to which it is also referred to as SARS-COV-2 (Peeri et al., 2020). It has been assessed that the virus spread mainly through infected respiratory droplets released from the cough or sneeze of the infected person. Though the virus is not generally airborne, evidence suggests that the infected droplets containing variable amounts of the virus can stay suspended in the air for some time (Rothan and Byrareddy, 2020).

Moreover, people can contract this virus by contacting infected surfaces through touching or other means (Repici et al., 2020). The global population is being told to wash their hands multiple times in a single day because the virus can remain viable on different surfaces for up to 72 h (Meyers et al., 2021). In most cases, the average incubation period of the virus is 5.2 days (Backer et al., 2020). However, there are significant variations that have been reported among individuals, especially in relevance to the onset and severity of symptoms. Moreover, many asymptomatic cases have been reported globally with varying transmission speeds (Mizumoto et al., 2020). The virus has spread rapidly in South Asian countries, including Pakistan.

It was on 26th February 2020 that Pakistan had reported its first two cases of COVID-19. As of 30th March 2020, there are a total of 1,717 confirmed cases of the novel coronavirus in Pakistan (Dawn.com, 2020). Since the emergence of this outbreak, the response efforts of the Pakistani Government have been swift. On 20th March, a partial lockdown was imposed in the Province of Sindh and on 23rd March 2020, in Punjab Province. It was around the same time that other provinces had imposed a lockdown in a bid to contain the spread of COVID-19 (Bol News, 2020). The lockdown, however, was not as swift and massive as seen in Wuhan, China, and other countries. As a result of this lockdown, many stayed at home and continued to isolate themselves socially. There have also been numerous accounts of food and mask shortages. In the healthcare sector, multiple reports have emerged regarding the shortage of protective equipment, medicine, and other forms of protective gear (Raza, 2020).

**TABLE 1:** Psychological impact scores of participants (*N* = 1361).

Variables	*N*	*%*
Impact of events		
Minimal impact	960	70.5
Mild impact	167	12.3
Moderate impact	36	2.6
Severe impact	198	14.5
Total	1361	100
Scores (IES-R)		
24 or more	960	70.5
33 or more	223	16.38
37 or more	189	13.88
Depression subscale		
Normal	942	69.2
Mild depression	112	8.2
Moderate depression	170	12.5
Severe depression	64	4.7
Extremely severe	73	5.4
Anxiety subscale		
Normal	882	64.8
Mild anxiety	85	6.2
Moderate anxiety	186	13.7
Severe anxiety	58	4.3
Extremely severe anxiety	150	11
Stress subscale		
Normal	1128	82.9
Mild stress	69	5.1
Moderate stress	67	4.9
Severe stress	58	4.3
Extremely severe stress	39	2.9

**TABLE 2:** Psychometric properties for Impact of Events Scale-Revised and DASS (Depression, Anxiety, and Stress Subscales) (*N* = 1361).

Variables	*M*	*SD*	Range	Cronbach’s α
Impact of events scale-R total score	19.19	16.72	0–88	0.95
DASS total score	11.06	12.99	0–126	0.96
Depression	22.14	4.70	0–42	0.91
Anxiety	3.60	4.37	0–42	0.86
Stress	3.71	4.59	0–42	0.89

M, mean; SD, standard deviation; α, Cronbach’s alpha.

Table shows the mean, standard deviation, Cronbach alpha value and response range. It was found that the Cronbach alpha values of the scales and sub-scales were in the good to excellent range (0.86–0.96).

**TABLE 3:** Regression of associations between demographic variables and scores on Impact of Events Scale-Revised and the DASS-21 Depression, Anxiety and Stress Subscales (*N* = 1361).

		Impact of events			Depression			Anxiety			Stress		
					
Variables	*n* (%)	*R* ^2^	*^A^R* ^2^	β [95% CI]	*R* ^2^	*^A^R* ^2^	β (95% CI)	*R* ^2^	*^A^R* ^2^	β (95% CI)	*R* ^2^	*^A^R* ^2^	β (95% CI)
Gender													
Male	456 (33.5)	0.01	0.01	−0.10* [−0.5.65, −1.91]	0.00	0.00	−0.01 [−1.38, 0.68]	0.00	0.00	0.03 [−1.62, 0.45]	0.00	0.00	−0.03 [−1.78, 0.33]
Female	905 (66.5)			Reference			Reference			Reference			Reference
Age (years)													
18–30	1086 (77.9)	0.01	0.01	0.08 [−1.72, 8.92]	0.01	0.00	0.08 [−1.16, 4.69]	0.00	0.00	0.07 [−1.15, 4.43]	0.00	0.00	0.04 [−1.98, 4.02]
31–40	177 (13.0)			0.04 [−3.78, 7.75]			0.06 [−1.33, 5.02]			0.08 [−0.73, 5.32]			0.08 [−0.96, 5.45]
41–50	59 (4.3)			−0.05 [−10.81, 2.65]			−0.05 [−6.32, 1.18]			−0.04 [−5.39, 1,67]			−0.04 [−1.08]
51–60	39 (2.9)			Reference			Reference			Reference			Reference
Marital status													
Single	949 (69.7)			0.18 [−17.08, 3.80]			−0.11 [−7.92, 3.47]			−0.15 [−8.44, 2.47]			−0.17 [−9.48, 2.25]
Married	389 (28.6)	0.00	0.00	0.22 [−18.75, 2.24]	0.00	0.00	−0.16 [−9.21, 2.33]	0.00	0.00	−0.19 [−9.27, 1.70]	0.00	0.00	−0.19 [−10.28, 1.79]
Divorced	13 (1.0)			−0.02 [−17.29, 10.27]			−0.01 [−9.11, 6.04]			−0.02 [−9.46, 4.97]			−0.01 [−8.96, 6.56]
Widowed	10 (0.7)			Reference			Reference			Reference			Reference
Educat-attainment													
Under-matriculation	8 (0.6)			0.07 [2.42, 31.43]			0.08 [1.74, 17.68]			0.10 [3.97, 19.10]			0.08 [2.33, 18.66]
Matriculation	22 (1.6)	0.00	0.00	0.07 [−0.85, 21.52]	0.01	0.00	0.05 [−1.88, 10.40]	0.01	0.01	0.05 [−1.82, 9.84]	0.00	0.00	0.06 [−1.20, 11.39]
Intermediate	87 (6.4)			0.14* [0.75, 19.60]			0.11 [−0.70, 9.65]			0.09 [−1.66, 8.11]			0.11 [−0.96, 9.65]
Graduation	635 (46.7)			0.26* [0.05, 17.74]			0.20 [−1.18, 8.53]			0.10 [−2.76, 6.43]			0.20 [−1.06, 8.89]
Post-graduation	538 (39.5)			0.19 [−2.06, 15.65]			0.12 [−2.55, 7.19]			0.03 [−4.01, 5.22]			0.14 [−2.19, 7.78]
Doctorate	57 (4.2)			0.19 [−2.06, 15.65]			0.05 [−2.98, 7.74]			0.03 [−3.77, 6.39]			0.07 [−2.19, 8.79]
Post-doctorate	14 (1.0)			Reference			Reference			Reference			Reference
Mother’s-educational attainment													
Under-matriculation	400 (29.4)			−0.05 [−14.47, 10.56]			−0.15 [−9.97, 3.79]			−0.17 [−9.88, 3.22]			−0.03 [−7.77, 6.31]
Matriculation	297 (21.8)	0.00	−0.00	−0.05 [−14.89, 10.22]	0.00	−0.00	−0.13 [−9.95, 3.85]	0.00	−0.00	−0.14 [−9.53, 3.61]	0.00	−0.00	−0.05 [−8.31, 5.81]
Intermediate	248 (18.2)			−0.01 [−13.40, 11.76]			−0.09 [−9.15, 4.68]			−0.13 [−9.60, 3.58]			−0.01 [−7.40, 6.75]
Graduation	306 (22.5)			−0.02 [− 13.53, 11.57]			−0.13 [−9.95, 3.84]			−0.14 [−9.57, 3.57]			−0.05 [−8.25, 5.86]
Post-graduation	103 (7.6)			−0.01 [−13.69, 11.95]			−0.07 [−9.53, 4.57]			−0.09 [−9.70, 3.73]			0.00 [−6.90. 7.51]
Doctorate	7 (0.5)			Reference			Reference			Reference			Reference
Father’s educational attainment													
Under matriculation	187 (13.7)			−0.00 [−10.45, 9.86]			0.02 [−4.96, 6.22]			−0.03 [−6.10, 5.54]			−0.03 [−6.06, 4.82]
Matriculation	231 (17.0)	0.00	0.00	−0.04 [−12.11, 8.10]	0.00	0.00	−0.01 [−5.97, 5.14]	0.00	0.00	−0.04 [−6.37, 4.22]	0.00	0.00	−0.09 [−7.96, 3.40]
Intermediate	224 (16.5)			−0.04 [−11.95, 8.27]			−0.03 [−6.33, 4.79]			−0.07 [−7.01, 3.58]			−0.10 [−8.27, 3.10]
Graduation	451 (33.1)			−0.10 [−13.89, 6.11]			−0.07 [−7.04, 3.95]			−0.13 [−7.70, 2.77]			−0.16 [−8.96, 2.27]
Post-graduation	216 (15.9)			−0.03 [−11.90, 8.34]			−0.01 [−6.02, 5.13]			−0.06[−6.73, 3.87]			−0.08 [−7.77, 3.61]
Doctorate	41 (3.0)			−0.06 [−17.36, 4.87]			−0.03 [−8.10, 4.13]			−0.05 [−8.54, 3.11]			−0.08 [−10.69, 1.81]
Post-doctorate	11 (0.8)			Reference			Reference			Reference			Reference
Profession													
Student	624 (45.8)	0.00	0.00	−0.01 [−3.30, 2.39]	0.00	0.00	−0.04 [−2.38, 0.74]	0.00	0.00	−0.02 [−1.91, 1.05]	0.00	0.00	−10* [−3.58, −0.38]
Working	569 (41.8)			−0.07 [−5.32, 0.42]			−0.10* [−3.57, −0.41]			−0.10*[−3.37, −0.36]			−0.13* [−4.20, −0.97]
None	168 (12.3)			Reference			Reference			Reference			Reference
Household size													
6 people or more	727 (53.4)			0.23 [−2.42, 18.47]			0.07 [−3.77, 2.51]			0.10 [−4.72, 1.25]			0.02 [−2.53, 3.89]
3 to 5 people	577 (42.4)	0.00	0.00	0.23 [−2.45, 18.38]	0.00	0.00	0.08 [−7.32, 4.19]			0.16 [−8.41, 2.53]			0.06 [−4.72, 7.06]
2 people	47 (3.5)			0.07 [−2.06, 9.33]			0.02 [−3.77, 2.51]			0.07 [−4.72, 1.25]			0.02 [−4.10, 7.66]
One person	10 (0.7)			Reference			Reference			Reference			Reference

*p < 0.05; CI, Confidence Intervals.

COVID-19 has brought about an intensive level of fear among the Pakistani population. Moreover, keeping in view the rising death toll worldwide and the impact of the lockdown on the population, it is imperative to assess mental health issues in society. Past research evidence has shown that many psychosocial and mental health issues are seen in individuals during outbreaks (Mak et al., 2010). Mental health experts believe that pandemics are not marked for being medical phenomena only; they have a considerable impact on humans and society at various levels, leading to disruptions (Warheit et al., 1996; Su et al., 2007). Research has identified panic, depression, stress, anxiety, and post-traumatic stress disorder resulting from pandemics (Xu et al., 2010; Okusaga et al., 2011). Moreover, sleep disturbances, lower levels of perceived health and other mental disorders (Ng et al., 2006). These mental health issues have been reported extensively in the case of COVID-19 as well (Liu et al., 2020; Qiu et al., 2020).

It was also found that those who were old and more educated were the more susceptible to have been exposed to a positive case of SARs and those concerned about their hygiene were likely to take more effective precautionary measures (Caballero-Anthony, 2005; Yeung and Fung, 2007). Concerning gender differences, Wu et al. (2005) reported that being female, having low financial independence, and old age was associated with a negative psychological impact of SARs and higher scores on depression, stress, and anxiety (Tan and Enderwick, 2006). Bonanno et al. (2008) had also identified that being female gender and old were risk factors toward poor mental health status (Wang et al., 2020).

Rodríguez-Rey et al. (2020a) analyzed the psychological impact and associated factors during the initial wave of the corona virus pandemic in Spain. The analysis of demographic factors including age, gender, education level, marital status, family income and province of residence were analyzed. Results showed that the psychological impact of corona virus pandemic decreased with age. In relevance to gender, females reported higher levels of psychological distress in comparison to males. In relation to education and socioeconomic status (assessment through family income), participants with a Ph.D. degree and those belonging to high and middle socioeconomic statuses showed lower psychological impact than groups with lower levels of educational attainment. Cortés-Álvarez et al. (2020) found that being a female, older age, low socioeconomic status and having lower levels of education was associated with adverse psychological effects (depression, stress, and anxiety). Shevlin et al. (2020) found that individuals with low income or experiencing loss of income, low educational attainment, older age, and living alone reported higher psychological trauma and adverse mental health effects due to the corona virus pandemic. Liu et al. (2020) found being female, having low educational attainment and living alone was associated with higher levels of post-traumatic stress, depression, and insomnia.

Presently, there is limited information available to assess the mental health and psychological impact of COVID-19 on the public in Pakistan. As this pandemic is known for being the first-ever major outbreak in Pakistan, no prior attempts have been made to investigate and assess the mental and physical health correlates of the psychological effects of COVID-19 in the general population in Pakistan. Consequently, there is a high level of uncertainty surrounding this pandemic in the country. Moreover, most of the past research evidence on viral outbreaks has focused on assessing the epidemiology of such diseases, modes of transmission, clinical characteristics and resulting manifestations, rates of transmission, precautionary measures and randomized control trials to determine the efficacy of vaccines.

### Purpose and objectives

The present study examined the mental and physical health correlates of the psychological impact of COVID-19 in the general population during the first wave of COVID-19 in Pakistan. Epidemiological data on the psychological impact and mental health status resulting from COVID-19 in the general population is limited, and therefore this warrants the need of conducting a comprehensive analysis. Moreover, how best to respond to challenges during the outbreak is still unknown. Furthermore, the study provides insights into the psychological impact of the outbreak and the need for healthcare professionals to enforce measures focused on providing counseling and therapeutic interventions to promote the well-being and mental health of the communities during such challenging times. Another main aim of the study is to assess the general population’s psychiatric morbidity and determine the risk factors associated with COVID-19.

### Hypotheses

(1) There would be a significant predictive association among socio-demographic variables, psychological impact and mental health status resulting from COVID-19. (2) Poor self-reported physical health would be significantly associated with adverse psychological impact and poor mental health status because of COVID-19.

The aforementioned relationships were hypothesized to assess the psychological impact of the pandemic and to determine the mental health of the public during the pandemic. It is pertinent to mention that there was an extensive uncertainty due to the magnitude of the pandemic at that time. Based on our understanding of the purpose of the study, most of the research focused on identification of the associations among the physical and psychological characteristics of the public and the potential epidemiology of suspected patients and the healthcare challenges. Furthermore, the researchers were unable to find any research articles or national community based sampling surveys assessing the psychological impact and physical correlates associated with the COVID-19 pandemic in the general population of Pakistan.

## Materials and methods

### Design

A cross-sectional survey design was used to assess the mental health status and psychological effects of COVID-19 via using an online questionnaire. Participants were recruited using a conveniently approached snowball sampling technique focused on the recruitment of the public living in Pakistan during the pandemic. Given the fact that no financial resources were available to the researchers and due to time sensitivity associated with the corona virus pandemic, we had preferred to use the snow ball sampling approach. It is also critical to note that the sampling strategy was not in accordance with a random selection of the sample. Participants were recruited through a strategy in which university students were first recruited through sharing the survey online. They were then asked to pass on the link to the survey to other participants. The link to the google forms survey questionnaire was shared through WhatsApp. The participants were also encouraged to share the link to the questionnaire via Facebook and Twitter. As the Pakistani Government had imposed a strict lockdown to prevent the spread of the corona virus and as the public was encouraged to minimize all forms of face to face interaction, potential participants had been invited to fill in the questionnaires electronically.

### Participants

This strategy allowed for the recruitment of a total of 1,361 participants from all over Pakistan. This sampling strategy and recruitment method were used in the face of a countrywide lockdown and to eliminate the probability of face-to-face interaction. The online survey was first shared with university students and then across a number of professional as well as informal networks of friends and family. All potential participants were encouraged to share the questionnaires with others.

#### Inclusion criteria

All individuals above the age range of 18 years of age had been encouraged to participate in the study. As the corona virus pandemic had a wide spanning impact on the entire population of the country and due to the imposition of a countrywide lockdown, maximum attempts were made to include a diverse sample.

#### Exclusion criteria

All participants below the age range of 18 were excluded from the sample. As the data collection was being done online and since children are unable to provide informed consent, it was decided that no participants below the age range of 18 will be included. The responses which were returned by individuals below this age range were deliberately excluded from the study. The researchers had designed the statement of informed consent at the beginning of the survey questionnaire to inform all participants below the age range of 18 not to participate in this research.

### Procedure

Potential respondents were asked to fill out the survey electronically. The survey platform used was “Google Forms,” widely used for such research endeavors. The researchers had thoroughly assessed the ethical considerations and followed all the protocols. Participants were ensured about their anonymity and confidentiality; purpose of the research was explained to the participants, and they were given rights if they wanted to withdraw at any time of study. Informed consent was sought from all participants through a consent form attached at the start of the questionnaire. Before being administered the questionnaire, their consent was sought by including informed consent at the beginning of the online questionnaire. Expedited ethical approval was sought through discussion among the Senior Faculty Members of the Department of Psychology including Senior Clinical Psychologists, Govt. College University, Lahore, in which no serious ethical risks were identified. Moreover, due to ethical requirements about anonymity and confidentiality, the participants were asked not to report their name and other identifying information. Therefore, there was no issue regarding any breach of their personal information. None of the research participants had raised any concerns about the study and had actively participated in data collection. Data collection for the study took place over 3 days, i.e., from 29th March 2020 to 31st March 2020, following the declaration of WHO in which COVID-19 had been declared a global pandemic. During this time, many potential participants were contacted and as all researchers had engaged in sharing the questionnaire with their contacts, a sufficient sample size was achieved during this time. The researchers had also planned that data collection via the online survey questionnaire will continue until a sufficient sample size will not be achieved. Moreover, the time duration was crucial for data collection in order to ensure novelty of research findings. However, other phases of the research planned continue until the completion of the research.

The sample size was calculated with a 95% confidential interval in accordance with a total of 220,892,430 population for Pakistan as of 2020. A total of 1,361 participants had completed the measures, which amounted to a 3% margin of error (Suresh and Chandrashekara, 2012).

### Measures

Previous surveys developed to assess the psychological effects and mental health status of individuals during the SARs outbreak were reviewed for the development of the survey questionnaire for the study (Rubin et al., 2010). The authors had also included questions pertaining to WHO Guidelines surrounding the current COVID-19 outbreak (World Health Organization, 2020). The questionnaire used was standardized and it was also confirmed that checking the alpha reliability used to assess the psychological impact and current mental health status of the participants. The psychological impact of the COVID-19 outbreaks, and current mental health status were assessed using the DASS-21 (Weiss, 2007) and Impact of Events Scale-Revised (Horowitz et al., 1979) both of which are standardized questionnaires. Moreover, the lockdown imposed in Pakistan and the guidelines from the government and healthcare agencies of the country were used to create additional questions related to the outbreak. As a result, a structured questionnaire was developed that covered several areas, including sociodemographic data. Most of the respondents were women (66.5)%, in the age range of 18–30 (77.9%), were single (69.7%), graduate (46.7%), and postgraduates (39.5%), students (45.8%), living with their families (93.8%) and in a household with six or more people (53.4%). 33.5% of the participants were males, a majority of whom were in the age range of 21–30 (75.2%), were single (67.8%), had at least graduate-level education (85.7%), were students (44.7%), and living with family (94%). In relation to the representation proportion of different cities of Pakistan, 45.4% of participants were from Lahore City, 6.8% from Karachi City, 4.2% from Sheikhupura City, 2.9% from Multan City, 2.4% from Gujranwala City, 2.2% from Sargodha and 2.4% from Rawalpindi and other regions. Thus, most of the participants sampled were from Punjab with lower representation from Sindh, Khyber Pakhtunkhwa and Baluchistan provinces. Other data included physical symptoms in the past 7 days (lockdown imposed on 23rd March 2020), diagnostic testing for COVID-19, protective measures, self-quarantine, precautionary measures taken at home and additional information deemed necessary to assess the demographic information surrounding the outbreak. The psychological impact of the COVID-19 outbreak was assessed through the usage of the Impact of Events Scale-Revised, and current mental health status was assessed DASS-21 item questionnaire.

Sociodemographic data were also collected on gender, age, marital status, years of schooling, parental educational attainment, profession, living arrangement, current residential location, testing for COVID-19 and household size. The self-reported physical health status of the participants in the past 7 days was determined through items designed to assess physical symptoms such as persistent fever, chills, headaches, breathing difficulty, dizziness, sore throat, persistent fever, and coughing and breathing difficulty. Knowledge about COVID-19 was assessed through data collection about precautionary measures taken at home, self-quarantine, and other protective measures.

#### Impact of Events Scale-Revised

Impact of Events Scale-Revised (IES-R) was used to assess the psychological impact of COVID-19. The IES-R is a self-administered questionnaire based on a five-point Likert scale developed by Weiss (2007). It is based on the original version of the Impact of Events Scale developed by Horowitz et al. (1979). It has been well-validated in the Pakistani population to document the psychological effects in earthquake recovery workers, emergency medical service personnel in the aftermath of terrorist suicide bombings and other public health crisis in Pakistan (Ehring et al., 2011; Razik et al., 2013; Kerai et al., 2017). The scale is designed to be used within 1 week of exposure to a public health crisis. The 22-item questionnaire comprises three subscales aimed at assessing mean avoidance, intrusion, and hyper-arousal (Asukai et al., 2002). It is primarily aimed at assessing the symptoms of PTSD and is not used as a diagnostic instrument (Motlagh, 2010). The respondents are required to score items from 0 to 3. The scoring range is from 0 to 88. On this test, the scores that exceed 24 are marked for being meaningful and clinically significant. The total IES-R score is divided into several domains. A score from 0 to 24 is considered normal, 24 to 32 is classified as mild (translates to a mild psychological impact). The score of 33 to 36 translates into a moderate psychological impact, and scores exceeding 37 indicate a severe psychological impact. The score range of 33 to 38 represents the cut-off for receiving a probable diagnosis of PTSD (Creamer et al., 2003). Moreover, the scores of 39 or higher are enough to result in a suppression of the immune system’s functioning even after 10 years following an impact event (Kawamura et al., 2001). The total alpha reliability of the scale is from 0.91 to 0.94, which indicates good internal consistency of the instrument.

#### Depression, Anxiety and Stress Scales

Depression, Anxiety and Stress Scales (DASS-21) by Lovibond and Lovibond (1995) was used to assess participants’ mental health status. It is a set of three self-report scales aimed at measuring the varying emotional states of depression, anxiety, and stress. Each of the scales comprises seven items and divided into Subscales containing similar content (Lovibond and Lovibond, 1995). The Depression Subscale is used for assessing dysphoria, self-depreciation, hopelessness, lack of interest/anhedonia and inertia. The Anxiety Subscale measures skeletal muscle effects, level of autonomic arousal, subjective experience of anxious affect and situational anxiety. The Stress Subscale is used for the measurement of chronic non-specific arousal. It also provides insights about difficulty relaxing, agitation, nervous arousal, impatience, and over-reactivity. Scores on the three subscales are measured through summing scores for the relevant items. DASS-21 has good alpha reliability values of 0.81, 0.89, and 0.78 for its three subscales (Clara et al., 2001; Osman et al., 2012). The instrument has also been found to exhibit commendable psychometric properties (Coker et al., 2018).

### Statistical analyses

Descriptive statistics were calculated and assessed keeping in view the sociodemographic characteristics, the nature, and types of physical symptoms along with variables such as contact history, knowledge about COVID-19, precautionary measures, and compliance with additional health guidelines. The percentages of responses had been analyzed in accordance with the total number of participants per response with specific emphasis on assessing the total number of responses given on each question. Furthermore, the scores attained on IES-R and DASS Subscales had been presented through descriptive statistics including the means and standard deviations. Several linear regressions were calculated to analyze the univariate associations and relationships among the sociodemographic variables, different physical symptoms, contact history, knowledge about the virus, compliance with precautionary guidelines and other related measures. The statistical tests used were two tailed in which the significance level was *p* < 0.05 as a standard convention. Statistical analyses were executed through SPSS 21.0.

## Results

A total of 1,361 respondents had completed the online questionnaire. The participants who had completed the questionnaire were selected for the study. Overall, 849 respondents had submitted the questionnaire on 29th March 2020, 445 respondents submitted their responses on 30th March, and 61 participants submitted their responses on 31st March 2020.

### Sociodemographic data and mental health

The results also showed that being male was significantly associated with lower scores on the Impact of Events Scale-Revised (β = −0.10, *p* < 0.05) but was not associated with DASS Subscale scores. In relevance to educational attainment, graduate-level education was significantly associated with the highest scores IES-R (β = 0.14, *p* < 0.05) followed by intermediate education (β = 0.14, *p* < 0.05). Concerning profession, working status was significantly associated with lower scores on DASS Depression Subscale (β = −0.10, *p* < 0.05), DASS Anxiety Subscale (β = −0.10, *p* < 0.05) and DASS Stress Subscale (β = −0.13, *p* < 0.05). Other socio-demographic variables, including age, marital status, parents’ educational attainment and household size, were not significantly associated with scores on the Impact of Events Scale-Revised or the DASS Subscale scores.

### Self-reported physical health and adverse mental health indicators

The physical health characteristics of the respondents have been presented in Table 4. The results also showed that having a persistent fever (>38°C) was significantly associated with higher scores on the DASS Anxiety Subscale (β = 0.06, *p* < 0.05), but no significant association was found for scores on the IES-R, the DASS Depression Subscale and the DASS Stress Subscale. The respondents who had chills were associated with higher scores on IES-R (β = 0.11, *p* < 0.05), DASS Depression Subscale (β = 0.12, *p* < 0.05), DASS Anxiety Subscale (β = 0.13, *p* < 0.05) and DASS Stress Subscale (β = 0.13, *p* < 0.05). Furthermore, having a headache was significantly associated with higher scores on the IES-R (β = 0.14, *p* < 0.05), the DASS Depression Subscale (β = 0.14, *p* < 0.05), DASS Anxiety Subscale (β = 0.14, *p* < 0.05) and DASS Stress Subscale (β = 0.14, *p* < 0.05). Breathing difficulty was significantly associated with higher scores on the IES-R (β = 0.13, *p* < 0.05), the DASS Depression Subscale (β = 0.18, *p* < 0.05), DASS Anxiety Subscale (β = 0.25, *p* < 0.05) and DASS Stress Subscale (β = 0.19, *p* < 0.05). Dizziness in respondents was also found to be significantly associated with higher scores on the IES-R (β = 0.22, *p* < 0.05), the DASS Depression Subscale (β = 0.19, *p* < 0.05), the DASS Anxiety Subscale (β = 0.23, *p* < 0.05) and DASS Stress Subscale (β = 0.20, *p* < 0.05). The symptoms of sore throat were significantly associated with higher scores on the IES-R (β = 0.17, *p* < 0.05), Depression Subscale (β = 0.16, *p* < 0.05), Anxiety Subscale (β = 0.20, *p* < 0.05), and Stress Subscale (β = 0.18, *p* < 0.05). Having a persistent cough, fever and breathing difficulty was significantly associated with higher scores on the IES-R (β = 0.11, *p* < 0.05), the DASS Depression Subscale (β = 0.12, *p* < 0.05), the DASS Anxiety Subscale (β = 0.13, *p* < 0.05) and Stress Subscale (β = 0.12, *p* < 0.05).

**TABLE 4:** Regression of associations between self-reported physical health status and scores on Impact of Events Scale-Revised and the DASS-21 Depression, Anxiety and Stress Subscales (*N* = 1361).

		Impact of events			Depression			Anxiety			Stress		
					
Variables	*n* (%)	*R* ^2^	*^A^R* ^2^	β [95% CI]	*R* ^2^	*^A^R* ^2^	β (95% CI)	*R* ^2^	*^A^R* ^2^	β (95% CI)	*R* ^2^	*^A^R* ^2^	β (95% CI)
Persistent fever (>38°C for at least 1 day)													
Yes	176 (12.9)	0.00	0.00	0.03 [−0.88, 4.41]	0.00	0.00	0.03 [−0.31, 1.14]	0.00	0.00	0.06* [0.18, 1.56]	0.00	0.00	0.02 [−0.38, 1.10]
No	1185 (87.1)			Reference			Reference			Reference			Reference
Chill													
Yes	156 (11.5)	0.01	0.01	0.11* [3.49, 9.03]	0.01	0.01	0.12* [1.98, 5.03]	0.01	0.01	0.13* [2.36, 5.26]	0.01	0.01	0.13* [2.37, 5.49]
No	1205 (88.5)			Reference			Reference			Reference			Reference
Headache													
Yes	326 (24)	0.02	0.01	0.14* [3.48, 7.60]	0.02	0.02	0.14* [2.01, 4.28]	0.02	0.02	0.14* [1.91, 4.07]	0.02	0.02	0.14* [2.08, 4.40]
No	1035 (76)			Reference			Reference			Reference			Reference
Cough													
Yes	241 (17.7)	0.02	0.02	0.16* [4.77, 9.37]	0.02	0.02	0.15* [2.54, 5.07]	0.02	0.02	0.17* [2.72, 5.12]	0.02	0.02	0.16* [2.70, 5.28]
No	1120 (82.3)			Reference			Reference			Reference			Reference
Breathing difficulty													
Yes	70 (5.1)	0.01	0.01	0.13* [6.40, 14.38]	0.03	0.03	0.18* [5.53, 9.88]	0.06	0.06	0.25* [7.91, 11.99]	0.03	0.03	0.19* [6.02, 10.46]
No	1291 (94.9)			Reference			Reference			Reference			Reference
Dizziness													
Yes	182 (13.4)	0.04	0.04	0.22* [8.37, 13.46]	0.03	0.03	0.19* [3.92, 6.74]	0.05	0.05	0.23* [4.57, 7.23]	0.04	0.04	0.20* [4.19, 7.07]
No	1179 (86.6)			Reference			Reference			Reference			Reference
Sore throat													
Yes	170 (12.5)	0.02	0.02	0.17* [5.93, 11.23]	0.02	0.02	16* [3.14, 6.06]	0.04	0.04	0.20* [4.05, 6.80]	0.03	0.03	0.18* [3.81, 6.78]
No	1191 (87.5)			Reference			Reference			Reference			Reference
Persistent fever, cough and breathing difficulty													
Yes	23 (1.7)	0.01	0.01	0.11* [8.60, 22.30]	0.01	0.01	0.12* [5.30, 12.83]	0.01	0.01	0.13* [5.80, 12.96]	0.01	0.01	0.12* [5.33, 13.03]
No	1338 (98.3)			Reference			Reference			Reference			Reference

*p < 0.05; CI, Confidence Intervals.

### Predictive association among awareness about COVID-19, psychological impact and mental health status

Table 5 shows the associations between awareness about COVID-19 and scores on the IES-R, DASS Depression, Anxiety and Stress subscales. The responses indicate that 1,330 (97.7%) of the respondents had not been tested for detection of COVID-19, 04 (0.3%) were tested and found positive, and 27 (2.0%) had been tested and were found negative. Among the respondents, 1275 (93.7%) had enforced protective measures at home, and 1,082 (79.5%) were in self-quarantine. Though a significant association was not found for COVID-19 testing (tested positive, tested negative or no test taken), but still it was found that only a small number of individuals had taken the test, i.e., 31 individuals out of 1,361 participants. Enforcement of protective measures at home was significantly associated with higher scores on the IES-R (β = 0.06, *p* < 0.05), but no significant associations were found on the other subscales of DASS. It was also found that being in self-quarantine was significantly associated with higher scores on the IES-R (β = 0.08, *p* < 0.05), the DASS Depression Subscale (β = 0.08, *p* < 0.05) and the DASS Anxiety Subscale (β = 0.05, *p* < 0.05), but with no significant associations for the DASS Stress Subscale.

**TABLE 5:** Regression of associations between awareness about COVID-19 and scores on the Impact of Events Scale-Revised and the DASS-21 Depression, Anxiety and Stress Subscales (*N* = 1361).

		Impact of events			Depression			Anxiety			Stress		
					
Variables	*n* (%)	*R* ^2^	*^A^R* ^2^	β [95% CI]	*R* ^2^	*^A^R* ^2^	β (95% CI)	*R* ^2^	*^A^R* ^2^	β (95% CI)	*R* ^2^	*^A^R* ^2^	β (95% CI)
COVID 19 testing													
No test taken	1330 (97.7)	0.01	0.01	0.01 [−5.23, 7.42]	0.01	0.01	0.01 [−2.83, 4.13]	0.01	0.01	0.00 [−2.81, 3.82]	0.01	0.01	0.01 [−2.78, 4.43]
Yes, tested positive	04 (0.3)			0.11 [17.23, 52.19]			0.11 [9.14, 28.37]			0.11 [9.18, 27.48]			0.11 [10.06, 29.74]
Yes, tested negative	27 (2.0)			Reference			Reference			Reference			Reference
Enforcement of protective measures													
Yes	1275 (93.7)	0.00	0.00	0.06* [0.58, 7.87]	0.00	0.00	0.00 [−1.87, 2.14]	0.00	0.00	0.00 [−2.45, 1.37]	0.00	0.00	0.00 [−1.85, 2.26]
No	86 (6.9)			Reference			Reference			Reference			Reference
Currently in self-quarantine													
Yes	1082 (79.5)	0.00	0.00	0.08* [1.28, 5.67]	0.00	0.00	0.08* [0.81, 3.23]	0.00	0.00	0.05* [0.08, 2.38]	0.00	0.00	0.04 [−0.20, 2.27]
No	279 (20.5)			Reference			Reference			Reference			Reference

*p < 0.05; CI, Confidence Intervals.

### Self-reported physical health and adverse mental health indicators

Table 6 shows the associations between demographic variables and self-reported physical health status with the IES-R Intrusion, Avoidance, and Hyperarousal Subscales. Results indicated that being male was significantly associated with lower scores on the IES-R Intrusion Subscale (β = −0.11, *p* < 0.05), the IES-R Avoidance Subscale (β = −0.08, *p* < 0.05) and the IES-R Hyperarousal Subscale (β = −0.10, *p* < 0.05). In terms of educational attainment, graduate respondents had the highest scores on the IES-R Intrusion Subscale (β = 0.22, *p* < 0.05) and the IES-R Avoidance Subscales (β = 0.31, *p* < 0.05). At the same time, no significant association was found in terms of the IES-R Hyperarousal Subscale. Enforcement of protective measures at home was significantly associated with higher scores on the IES-R Intrusion Subscale (β = 0.05, *p* < 0.05), the IES-R Avoidance Subscale (β = 0.05, *p* < 0.05) and the IES-R Hyperarousal Subscale (β = 0.06, *p* < 0.05). Results also showed that being currently in self-quarantine was significantly associated with higher scores on the IES-R Intrusion Subscale (β = 0.08, *p* < 0.05), the IES-R Avoidance Subscale (β = 0.08, *p* < 0.05) and the IES-R Hyperarousal Subscale (β = 0.06, *p* < 0.05).

**TABLE 6:** Regression of associations between demographic variables and Impact of Events Scale-Revised Intrusion, Avoidance and Hyperarousal Subscales (*N* = 1361).

		Intrusion			Avoidance			Hyper−arousal		
				
Variables	*n* (%)	*R* ^2^	*^A^R* ^2^	β [95% CI]	*R* ^2^	*^A^R* ^2^	β (95% CI)	*R* ^2^	*^A^R* ^2^	β (95% CI)
Gender										
Male	456 (33.5)	0.01	0.01	−0.11* [−2.23, −0.79]	0.00	0.00	−0.08* [−1.93, −0.47]	0.01	0.01	−0.10* [−1.60, −0.51]
Female	905 (66.5)			Reference			Reference			Reference
Educat-attainment										
Under-matriculation	8 (0.6)	0.00	0.00	0.07 [0.59, 11.75]	0.00	0.00	0.07 [0.35, 11.61]	0.00	0.00	07 [0.52, 9.00]
Matriculation	22 (1.6)			0.05 [−1.37, 7.23]			0.10 [0.97, 9.65]			0.05 [−1.17, 5.35]
Intermediate	87 (6.4)			0.12* [−0.26, 6.99]			0.17* [0.89, 8.20]			0.11 [−0.48, 5.02]
Graduation	635 (46.7)			0.22* [−0.49, 6.31]			0.31* [0.72, 7.58]			0.18 [−0.75, 4.42]
Post-graduation	538 (39.5)			0.16 [−1.23, 5.53]			0.24 [−0.19, 6.67]			0.14 [−1.16, 4.01]
Doctorate	57 (4.2)			0.10 [−1.28, 7.23]			0.10 [−0.45, 7.11]			0.08 [−0.90, 4.80]
Post-doctorate	14 (1.0)			Reference			Reference			Reference
COVID-19 testing										
No test taken	1330 (97.7)	0.01	0.01	0.01 [−1.93, 2.94]	0.00	0.00	0.01 [−1.83, 3.10]	0.01	0.01	0.00 [−1.90, 1.79]
Yes, test positive	04 (0.3)			0.11 [6.84, 20.28]			0.09 [4.69, 18.29]			0.10 [4.55, 14.76]
Yes, tested negative	27 (2.0)			Reference			Reference			Reference
Enforcement of protective measures										
Yes	1275 (93.7)	0.00	0.00	0.05* [0.10, 2.91]	0.00	0.00	0.05* [0.01, 2.85]	0.00	0.00	0.06* [0.22, 2.35]
No	86 (6.9)			Reference			Reference			Reference
Currently in self-quarantine										
Yes	1082 (79.5)	0.00	0.00	0.08* [0.46, 2.15]	0.00	0.00	0.08* [0.54, 2.24]	0.00	0.00	0.06* [0.13, 1.41]
No	279 (20.5)			Reference			Reference			Reference

*p < 0.05; CI, Confidence Intervals.

## Discussion

The present study aimed to assess the mental and physical health correlates of the psychological impact of COVID-19 on the mental health of the Pakistani population during the first wave. Results showed that participants who reported physical symptoms such as headache, chills, fever, breathing difficulty, dizziness, sore throat, persistent fever, cough and breathing difficulty showed higher levels of stress, depression and anxiety. The literature documenting the psychological effects of the first wave of the corona virus pandemic has also shown that individuals who experienced physical health symptoms linked with the corona virus reported adverse mental health effects (Cascella et al., 2020).

It was also found that almost one-third of the population surveyed reported an adverse psychological impact assessed through the IES-R. At the same time, one-fourth of the population exhibited a poor mental health status assessed through the DASS-21. The existence of these moderate to severe level psychiatric morbidities warrant the need for immediate psychological interventions. Consequently, this indicates an overall significant psychological impact of COVID-19 on the general population in Pakistan. Based on these findings that the pandemic has led to numerous psychological repercussions in the country. These findings are consistent with studies that have identified a wide range of psychological costs directly associated with similar outbreaks. Xiang et al. (2020) reported that COVID-19 has parallels with the 2003 SARs outbreak during which many of those infected had reported extreme stress, depression, anxiety, a sense of isolation, fear, and stigma. Wu et al. (2005) had reported adverse psychological effects of SARs, a disease like COVID-19. It was found that depression, stress, and anxiety were commonly reported outcomes in the population. Other research evidence has also documented psychiatric morbidities in the population during and after outbreaks (Su et al., 2007; Lau et al., 2008; Mak et al., 2009).

The present study’s findings have identified that most of the females reported a moderate to severe psychological impact of the pandemic. Specifically, being female was likely to be associated with higher scores on the IES-R Subscales, including Intrusion, Avoidance and Hyperarousal. These findings also show that females are more likely to experience memories of the event, COVID-19. Moreover, they are likely to engage in attempts to avoid reminders of the event. They are also more likely to show a heightened sense of uncontrolled alertness whenever they would be reminded about the event. It also shows that being male might be a protective factor to an adverse psychological impact and poor mental health status.

This finding is consistent with the research showing that females are more likely to respond adversely and report a higher prevalence of psychological problems during outbreaks and other natural disasters (Warheit et al., 1996; Mak et al., 2010; Ehring et al., 2011; Warsini et al., 2014). The literature has also shown that females are at a higher risk and show higher global prevalence rates of depression compared to their male counterparts (Karger, 2014; Albert, 2015). Due to this natural pattern of being more susceptible to depression, the present study results warrant immediate psychological support interventions for females.

The socio-demographic data also indicates that being a graduate (having 16 years of education) was also significantly associated with an adverse psychological impact and poor mental health status exhibited through higher scores on depression, stress, and anxiety. One possible explanation might be that the educated participants have more awareness about the devastating physical effects of the disease and the speed of transmission, due to which the higher scores were observed. Moreover, a higher level of social media exposure and more extensive information and awareness about COVID-19 might be responsible for the adverse psychological impact and poor mental health status. Cheng et al. (2005) that higher education is associated with avoidance symptoms and distress. Bauldry (2015) had studied the protective effect of education on mental health. The findings showed much variation to whether higher educational attainment protects against adverse psychological effects. However, most research evidence does suggest that individuals who have higher educational attainment have better mental health status and less prone to an adverse psychological impact of major life events (Hall et al., 2008; Mak et al., 2010).

Another important socio-demographic correlation identified was working status. The results show that individuals who were working exhibited lower scores on depression, stress, anxiety, and an overall lower psychological impact of COVID-19 than those who were currently studying or neither working and nor studying. This might point toward the economic side of COVID-19, with individuals who are working and consequently having more financial independence being more resilient toward the effects of this outbreak. The relevant literature on this area has also found that working status indicates individuals’ economic well-being, which in turn enhances their preparedness and effective psychological responses during outbreaks (Cranford, 2020). However, evidence suggests that employed individuals report more severe economic and psychological effects during outbreaks, especially when they do not have a stable line of employment (Wen et al., 2005).

The present study also indicated a significant association among self-reported physical health, psychological impact, and current mental health status. It was found that having a persistent fever, chills, headache, cough, breathing difficulty, dizziness, and sore throat was positively associated with an adverse psychological impact of COVID 19 in addition to depression, stress, and anxiety. As individuals with physical symptoms most associated with COVID-19 exhibited higher levels of depression, stress, anxiety, and adverse psychological impact, it is imperative for mental health professionals and governmental authorities to provide immediate supportive interventions. Lack of psychological support and proper guidance about these symptoms and their overall self-reported physical health status can worsen their mental health functioning. In addition, the symptoms and psychological effects might exacerbate due to extensive social media coverage surrounding the possible symptoms of this outbreak. Cranford (2020) had drawn upon the work from previous outbreaks (Ebola, H1NI) and other forms of collective trauma, such as terrorist attacks where a higher level of media coverage of such events had unintended consequences for those at high risk as well as for those at risk of contracting the disease. Thus, these results suggest that individuals in the general population who have physical symptoms are at a higher risk of experiencing or being diagnosed with psychiatric morbidities in comparison to the rest of the population, a finding that is consistent with the relevant literature (Tan and Cheong, 2003; Hall et al., 2008; Lin et al., 2010).

Consequently, they are more likely to experience a weakened immune system response due to the psychological effects such as fear of being diagnosed with COVID-19 (Reiche et al., 2004; Won and Kim, 2016; Robson et al., 2017). The global wave of fear surrounding this outbreak might also explain the current mental health status of those who reported physical symptoms associated with the pandemic.

It was also found that awareness about this pandemic was significantly associated with adverse psychological effects. Specifically, a significant positive association was reported between enforcement of protective measures at home and reporting higher event-specific distress as measured through IES-R. However, this distress can be explained as an outcome of taking a wide range of precautionary measures to protect themselves and their families from being infected (Park and Park, 2020). Another possible explanation of the psychological impact of COVID-19 on the general population and resulting protective measures taken by them can be their first-time exposure to a global pandemic in the country. Another reason can be a lack of understanding surrounding the efficacy of protective measures that individuals have implemented at their homes.

The present study also highlighted that self-quarantine was also associated with higher scores on depression, stress, anxiety, and event-specific distress. These psychological effects might be due to uncertainty surrounding the overall global health landscape, fear of diagnosis, paranoia surrounding the pandemic that has been socially constructed due to excessive media discourse and coverage, and other existing psychiatric issues in the general population. The literature on this area has highlighted that large scale quarantine measures imposed by governments during outbreaks lead to adverse psychosocial effects on the population (Dong and Bouey, 2020). Ornell et al. (2020) had reported that being in quarantine can lead to varying levels of depression, anxiety, panic attacks and even psychotic episodes and suicide in individuals quarantined because of a positive diagnosis of COVID-19 (Brooks et al., 2020).

The study, however, has some limitations. First, the researchers did not use a random sampling technique and had to resort to snowball sampling due to time constraints. In addition, self-reported psychological impact and mental health status might not offer a clear reflection of the actual diagnosis offered by mental health professionals. The study did not document those participants who had actual contact history with individuals diagnosed with COVID-19. The study could sample a minimal number of participants who were diagnosed with COVID-19. As COVID-19 is a natural disaster and can also be marked for being a complex event with a wide range and multi-level psychosocial outcomes, the ascertainment of a causal relationship cannot be done based on the evidence gathered in the present study. Apart from these limitations, our study provides valuable insights into the mental health status of the general population in Pakistan and its association with self-reported physical health and sociodemographic correlates.

It is critical to note that the COVID-19 pandemic is continuing to spread globally. The present research findings can guide the development of immediate psychological support strategies and interventions aimed at promoting the mental health and well-being of the global population. Moreover, as the pandemic is ongoing, it is imperative to promote the preparedness of healthcare systems around the globe and to enhance the readiness of the governmental authorities and healthcare professionals in Pakistan in case a widespread transmission occurs in the country.

## Conclusion

The study showed that a significant amount of the population had a moderate to severe psychological impact during the first wave of the COVID-19 outbreak. It was also found that about one-fourth of the participants moderate to severe levels of depression, stress, and anxiety. Being female, being a graduate and having unstable employment is positively associated with moderate to adverse psychological impact and poor mental health status. Another important finding is that having self-reported physical symptoms (persistent fever, chills, headache, cough, breathing difficulty, and sore throat) were positively associated with higher scores on depression, stress, anxiety, and event-specific distress. The adverse psychological effects of being in self-quarantine were also documented in the study. We conclude with the recommendations for researchers, governmental entities, and public health professionals to provide immediate psychological support interventions to the general population, especially those reporting physical symptoms. As COVID-19 is the most significant public health crisis in Pakistan’s history and most possibly the globe, the government and healthcare professionals should focus on receiving and providing strategic and effective communications focused on promoting the populations’ physical and mental health functioning. It is also recommended that psychotherapies tailored specifically to treat psychological issues associated with COVID-19 need to be developed to promote individuals’ mental health status. It needs to be understood that COVID-19 is a multi-level and complex natural disaster and event presenting one of the greatest global health crises in the modern world’s history. This calls for the need for a global effort and research-based psychosocial and physiological health interventions to combat this pandemic. Also, when the COVID-19 pandemic is brought under control, psychological support mechanism and efforts will need to be focused on combating a probable mental health crisis that can be expected to emerge soon after the pandemic.

## Data availability statement

The original contributions presented in the study are included in the article/supplementary material, further inquiries can be directed to the corresponding author.

## Ethics statement

The studies involving human participants were reviewed and approved by the Institutional Bioethics Committee of the Government College University, Lahore. The patients/participants provided their written informed consent to participate in this study.

## Author contributions

SK collected all the data and worked out the statistical data, while CL and SH are in the role of supervisory assistance. RI proofread and suggested revisions. MF guided in the analysis and results and discussion. FA suggested revisions from a technical point of view and provided the references. All authors contributed to the article and approved the submitted version.
